# Expanding the field: using digital to diversify learning in outdoor science

**DOI:** 10.1186/s43031-022-00047-0

**Published:** 2022-03-10

**Authors:** Bethan C. Stagg, Justin Dillon, Janine Maddison

**Affiliations:** 1 Field Studies Council, Shrewsbury, Shropshire UK; 2 Graduate School of Education, University of Exeter, Exeter, UK; 3 School of Natural and Environmental Sciences, Newcastle University, Newcastle, UK

**Keywords:** Geography education, Outdoor learning, COVID-19, Fieldwork, Science education, Technological pedagogical content knowledge (TPCK)

## Abstract

This is an empirical study of teacher experiences with school learners (7–18 years) engaging in cross-curricular environmental science during the COVID-19 pandemic. The study focuses on #FieldworkLive, a programme of live-streamed outdoor science lessons produced by the Field Studies Council and Encounter Edu during the UK lockdown (April – May 2020). The experiences of approximately 377,000 teachers and students from 32 countries were captured using an online survey and direct staff consultation. This delivery method allowed us to reach untapped audiences and to provide learners with a virtual fieldwork experience during the constraints of lockdown. Teachers were highly positive about the technology-enhanced learning which provided them with novel perspectives and approaches for the classroom. We propose a model for the affordances provided by this delivery approach based on the Technological Pedagogical Content Knowledge framework. The Field Studies Council has developed a flexible package of multimedia resources for secondary schools as a route to enriching outdoor experience and learning despite the constraints imposed by the pandemic.

**Supplementary Information:**

The online version contains supplementary material available at 10.1186/s43031-022-00047-0.

## Introduction

The Field Studies Council (FSC) is a UK environmental education charity and a leading provider of science, geography, and cross-curricular learning outside the classroom (FSC, 2020. Prior to the pandemic, up to 3000 schools visited its 27 learning locations each year, including 25% of all A level (16–18 years) biology students in the UK. FSC’s teaching and learning focuses on the topics in UK science and geography curricula pertaining to the outdoor environment, for example the biotic and abiotic components in an ecosystem. FSC’s ethos is based on an interdisciplinary approach that encompasses affective and embodied learning as well as progress in the cognitive domain. This paper looks at the impacts of the FSC’s digital learning programme during the pandemic.

### Transitioning from outdoor to virtual

**Fig. 1:**
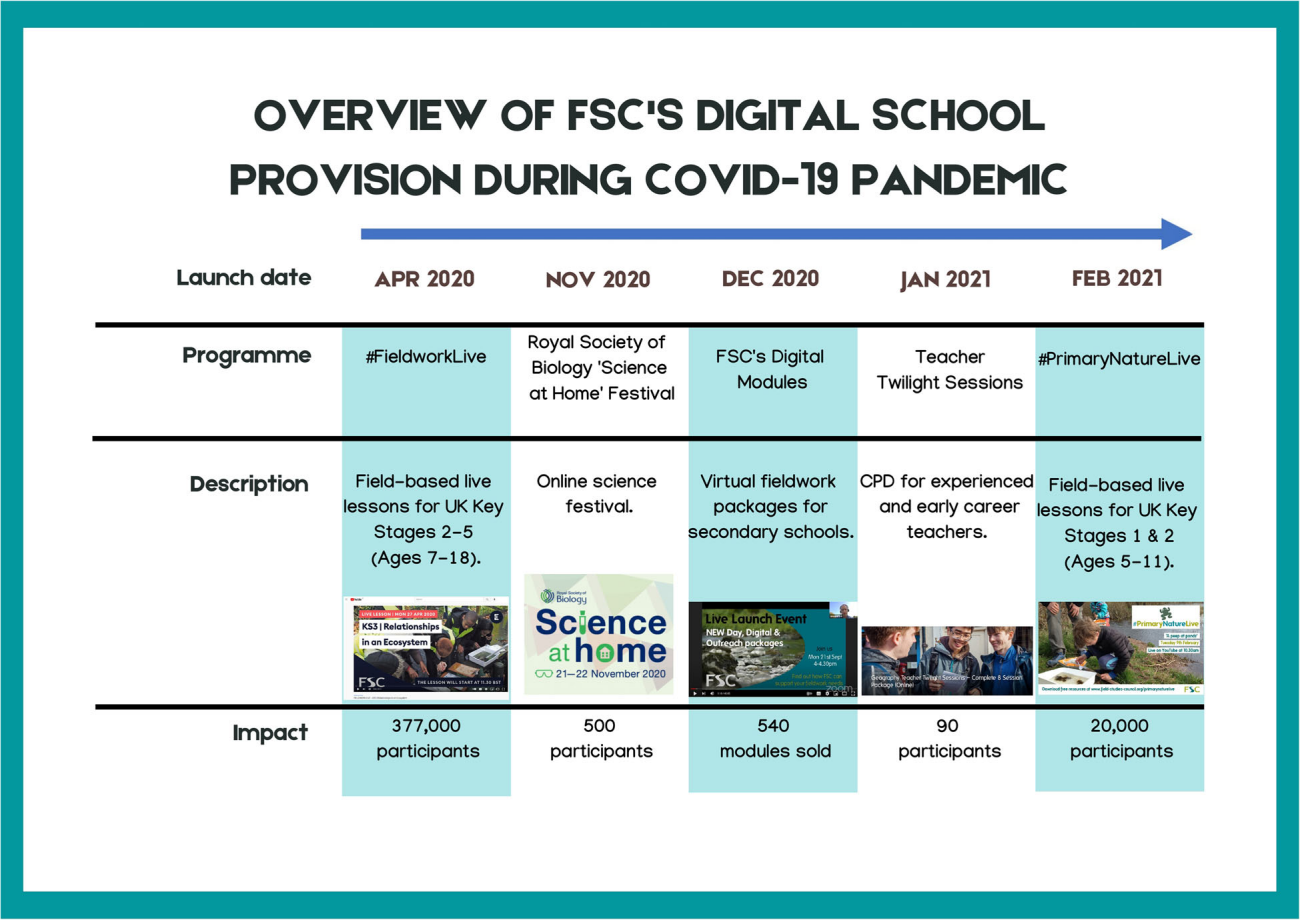
Overview of FSC’s digital school provision during the COVID-19 pandemic

**Fig. 2:**
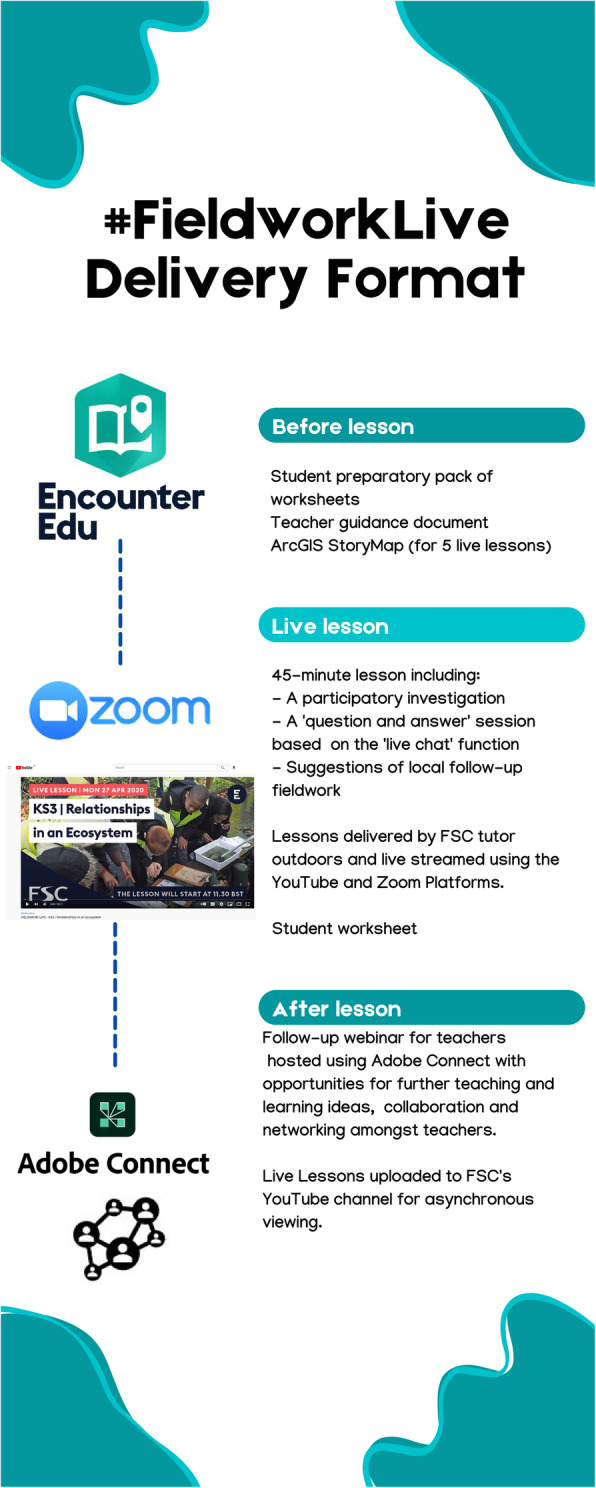
Typical delivery format for the #FieldworkLive sessions (Live lessons can be viewed by visiting the #FieldworkLive playlist on the FSC YouTube channel)

Like many educational institutions, the COVID-19 pandemic and lockdown constraints required the FSC to make a rapid transition from a predominantly ‘in-person’ communication mode to an online one (Engelbrecht et al., 2020) but with the additional challenge of being an outdoor education provider. FSC has supported teachers during the pandemic with a range of synchronous and asynchronous digital courses (Fig. 1), but this study will focus on #FieldworkLive, a comprehensive package of 10 live-streamed lessons (hereafter called ‘live lessons’) and accompanying resources, which took place in outdoor locations during April – May 2020 (Fig. 2). #FieldworkLive targeted 7–18-year-olds and had three pedagogic aims: (1) to enhance scientific and geographic knowledge, (2) to develop critical thinking skills, and (3) to inspire learners about related careers and environmental stewardship. The programme was designed and delivered by FSC’s tutors and other staff, with production support from digital educational company Encounter Edu (https://encounteredu.com/). #FieldworkLive was accessed by an estimated 377,000 teachers and students from 3089 schools and 32 countries (Encounter Edu, 2020).

More than 15,000 learners accessed the videos on the FSC’s YouTube channel after the live event (https://www.youtube.com/user/FieldStudiesCouncil), and the benefits of employing both synchronous and asynchronous modes in computer-mediated communication are discussed elsewhere (for example, Johnson, 2008).

A key strength of the technology-embedded format employed was that it did not require teachers to have technical expertise or specialist equipment, which are known to be key barriers to digital adoption (Bingimlas, 2009; Wang et al., 2014). In addition, the delivery mode did not require the remote-working students to have technical expertise since, contrary to popular assumptions, young people have limited digital skills (Bolaños & Salinas, 2021; Wang et al., 2014). The project also served to provide teachers with digital teaching support and an opportunity for collaboration, at a time when it was most needed (Engelbrecht et al., 2020).

### Pedagogic framework

The aim of #FieldworkLive was to provide a participatory online learning opportunity that promoted learners’ engagement with nature during the school closures and lockdown restrictions. Maintaining a focus on pedagogy not technology has been found to be crucial in the successful adoption of digital technologies (Burden & Atkinson, 2007; Shinneman et al., 2020; Welsh et al., 2013). To ensure ease of adoption, digital pedagogic frameworks underpinned the 3-step digital delivery format (Fig. 2).

Drawing upon Puentedura’s (2006) four-step SAMR model for digital technology adoption, #FieldworkLive did not simply aim to enhance existing fieldwork by substituting the mode of delivery to a digital format, but rather to modify fieldwork learning opportunities and tasks to transform the fieldwork experience. For example, #FieldworkLive enabled students to collect local field data using the ESRI app Survey 123 on their phones, to upload the data to the national dataset and examine these multi-site maps using ArcGIS. Students regularly used the Survey 123 app during FSC field courses before the pandemic but did not have the opportunity to explore their data in the context of a national primary dataset.

One of the criticisms of the SAMR model is its focus on technological tools, processes, and final product, without an assessment of the pedagogical aspects (Hamilton et al., 2016). For this reason, the production team used Koehler and Mishra’s (2005) Technological Pedagogical Content Knowledge (TPCK) framework for the development of the #FieldworkLive’s pedagogical focus and content. The TPCK framework, which built on Gudmundsdottir and Shulman’s (1987) Pedagogic Content Knowledge framework, sought to explain how the three areas of knowledge interact and could be applied to the effective integration of technology in learning. In #FieldworkLive, the aim was to use the TPCK principles to create a quality programme with high relevance and value to teachers. 

Producers shared the potential connections and affordances between and among content, pedagogy, and technology with the target audience through the ‘teacher guidance’ documents that supported the live lessons. A list of curricular-linked learning objectives and outcomes were developed for each lesson, an integral part of the instructional design process (Hamilton et al., 2016). The guidance included lesson plans, preparatory resources, worked examples and exemplar answers. Teachers were referred to further subject reading and student resources. Technical guidance on using the live lessons with remote classes was included and troubleshooting support was provided to teachers via email and social media. Teacher webinars were scheduled after the live lessons, as a method of facilitating learners’ understanding further through extension activities and as an opportunity for networking, sharing and collaboration. 311 teachers attended 37 free CPD ‘follow-up’ webinars (Encounter Edu, 2020). The low numbers (compared to live lessons) could reflect the constraints on the time available for teacher CPD during the pandemic. Teachers were also referred to resources as part of Encounter Edu’s professional development provision (https://encounteredu.com/cpd).

**Fig. 3:**
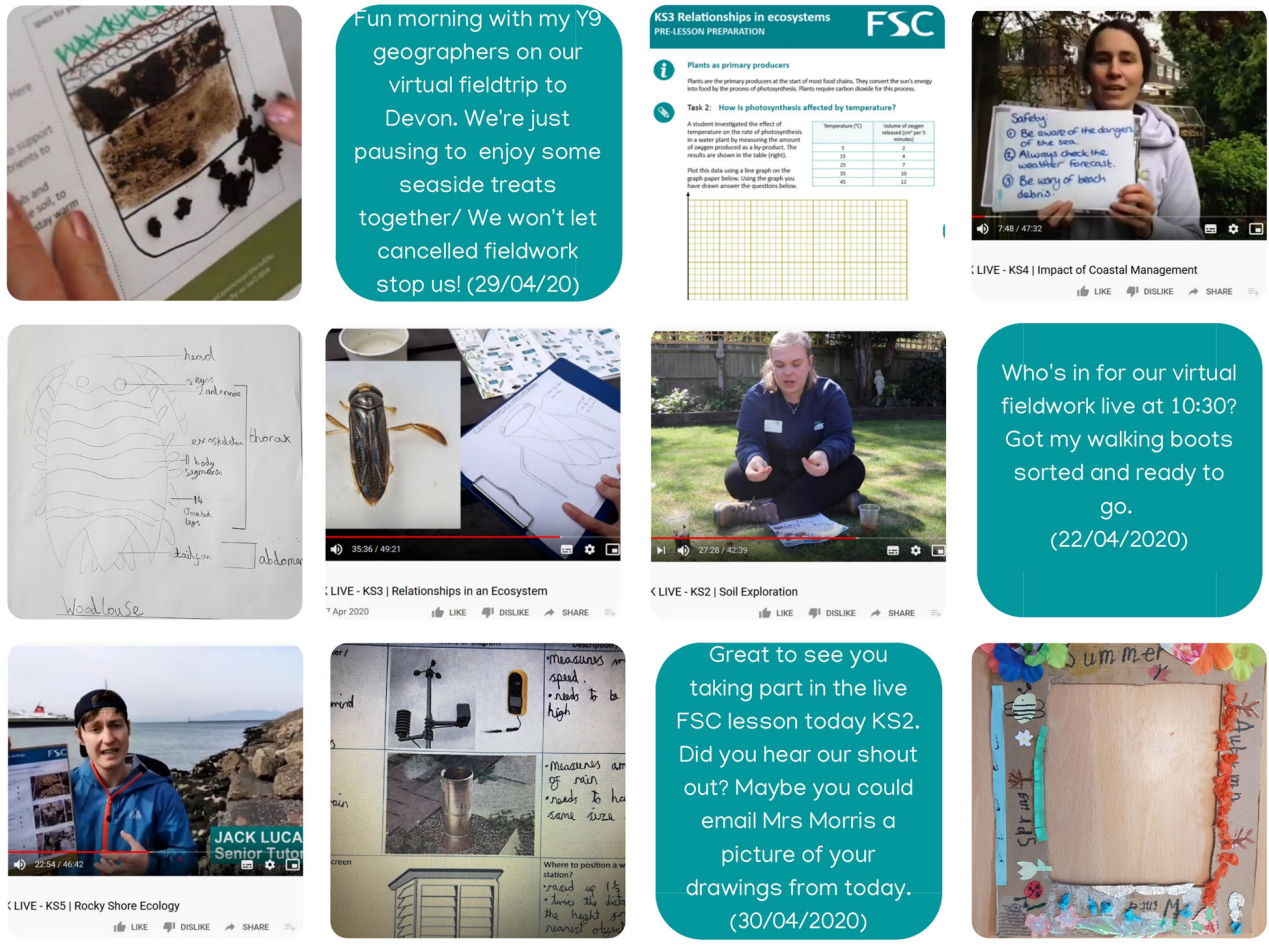
Examples of video content, student work and Tweets shared via #FieldworkLive

Educators often focus on the benefits of fieldwork for developing technical or subject-specific skills development but, as an inherently social activity, fieldwork also contributes substantially to learners’ interpersonal skills (Boyle et al., 2007). Virtual fieldwork experiences can also be designed to promote social learning (for example, Atchison et al., 2019), based on the social constructivist model of collaborative meaning making (Moreillon, 2015). The production team used ‘shout-outs’, live question-and-answer sessions and dialogue on social media channels via the project’s hashtag to develop the learner community for #FieldworkLive (Fig. 3). Sustaining the learner community was a challenge throughout #FieldworkLive, due to the high registration/attendance numbers, coupled with low staffing numbers. Also, at the point of delivery (April/May 2020) it can be assumed that this mode of learning and interaction was novel to the learner, who had limited experience of navigating digital communication and broadcast tools in a home environment. Learners need sufficient time and opportunities to become confident using digital communication media, to ensure that these media serve to develop a learner community, instead of just acting as a distraction (Pizzi, 2014).

The use of preparatory digital resources prior to instruction is a well-known approach in the ‘flipped classroom’ model (Enfield, 2013) and is becoming increasingly important in outdoor environmental science (for example, Remmen & Frøyland, 2015). Technology enhanced learning brings various well-documented benefits to fieldwork (Fletcher et al., 2007; Maskall et al., 2007; Cotton & Cotton, 2009; Welsh et al., 2013). First, it enables learners to familiarise themselves with field locations and conditions, thus reducing the novelty associated with the fieldwork environment and avoiding ‘cognitive overload’ in learners. Technology enhanced learning can be used to deliver elements of learning that are not dependent on in-person instruction in the outdoor environment. It allows learners to practise data collection techniques, both virtually and in local outdoor environments. Finally, technology enhanced learning is shown to be effective for developing students’ specialist and transferrable skills.

### Research objectives

A key strength of this study is that it focuses on teacher perceptions and course structure, as opposed to learner engagement, in contrast to many digital learning studies (Martin et al., 2020). The study investigates course design, development, and instructional characteristics. The target audience for the course was school learners, whereas most studies focus on university students (ibid.). Our research questions were:

1. What were the successful and unsuccessful aspects of the technology-embedded teaching and learning, as perceived by the instructors and participating teachers? What were teachers’ attitudes to the technology-embedded teaching and learning, and how have these changed because of #FieldworkLive?
2. What were the affordances and constraints of this type of technology-embedded teaching and learning, for the delivery of environmental science education during COVID-19?
3. What were the advantages of the technologies used by FSC because of COVID-19 that we should continue to practice in and out of classroom learning?

We will examine these questions using data from a sample of teachers who participated in #FieldworkLive, the results of an internal consultation, and a review of FSC’s digital developments since February 2020.

## Methods

We employed a sequential mixed methods approach, with questionnaires and a group interview to investigate the variables of interest in depth. The research was undertaken based on BERA’s (2018) Guidelines for Educational Research.

### Teachers’ perspectives

#### Participants and data collection

An online questionnaire was circulated to participating schools immediately after #FieldworkLive took place and was completed by 233 teachers from 26 April to 11 May 2020. The questionnaire was produced on the online survey site Survey Monkey (www.surveymonkey.com) and consisted of a total of six ‘closed’ and eight ‘open-ended’ questions. Eight questions investigated the perceptions and experiences of teachers and their students during #FieldworkLive and were analysed to help answer research question 2. The authors agreed that the remaining questions were not relevant to this enquiry, and they were omitted from the analysis. A list of questions is included in the Additional file 1.

#### Data analysis

The data for the three relevant open-ended questions were analysed using a thematic content analysis with no a priori categories. The first author developed the initial categories through immersion in the data and a preliminary coding exercise. Categories were refined through immersion in the data and subsequent discussion between two of the authors. These two authors then coded the response data independently and compared scores. Where the two coders’ scores differed by three points or more (which occurred for 10 out of 26 categories), authors compared individual coded segments and refined the coding and category definitions a second time. Inter-coder agreement was assessed using Freelon’s (2013) reliability calculator for Krippendorff’s Alpha (Hayes & Krippendorff, 2007). Krippendorff’s alpha was 0.94, which indicates a very high level of inter-assessor agreement for interval or ratio data (De Swert, 2012).

Categories were coded for all three questions but then each category was assigned to a particular question, according to where the frequency of responses was the highest. We took this action because a fault in the questionnaire design meant that we were unable to identify where respondents had repeated the same comment in different questions so risked pseudo-replication if responses were combined across questions. This fault occurred because Survey Monkey does not generate unique identifiers for each respondent but produces unique identifiers for the responses instead.

### Organisers’ perspectives

#### Participants and data collection

Six #FieldworkLive organisers (including the third author) participated in an internal consultation from 15 July to 16 September 2021. The consultation consisted of an online questionnaire with six open-ended questions about organisers’ perceptions of the technology enhanced learning (research questions 1 and 2) and a one-hour group interview by video conference about the educational implications of the technologies used (research question 3). The non-participating authors facilitated the group interview, which was guided by five leading questions, based on the key emerging themes in the questionnaire data. The list of questions for the questionnaire and interview are included in the Additional file 1.

#### Data analysis

Questionnaire data was analysed using constant comparison analysis, a common approach for generating a set of themes from human response data (Leech & Onwuegbuzie, 2007, 2008). The first author arranged the data into smaller segments after immersion in the data, grouped segments into similar categories and developed descriptors for the different categories.

The group interview data analysis comprised a thematic content analysis combined with an assessment of the level of consensus in the group, drawing on the micro-interlocutor approach developed by Onwuegbuzie et al. (2009) for focus group research. The analysis takes the interactive nature of focus group data into account, providing a deeper understanding of the dataset than traditional approaches to content analysis.

The second author assessed the initial questionnaire and interview analyses and indicated any points of disagreement. Participants were asked to check whether they agreed that the descriptors were accurately describing their statements, as a measure of descriptive validity. The revised analyses are presented as narrative accounts below. The summative data analyses are shown in the Additional file 1.

## Results

The quantitative and qualitative findings are organised alongside the research questions and presented using descriptive statistics and narrative accounts.

### What were the successful and unsuccessful aspects of the technology-embedded teaching and learning, as perceived by the instructors and participating teachers?

**Fig. 4:**
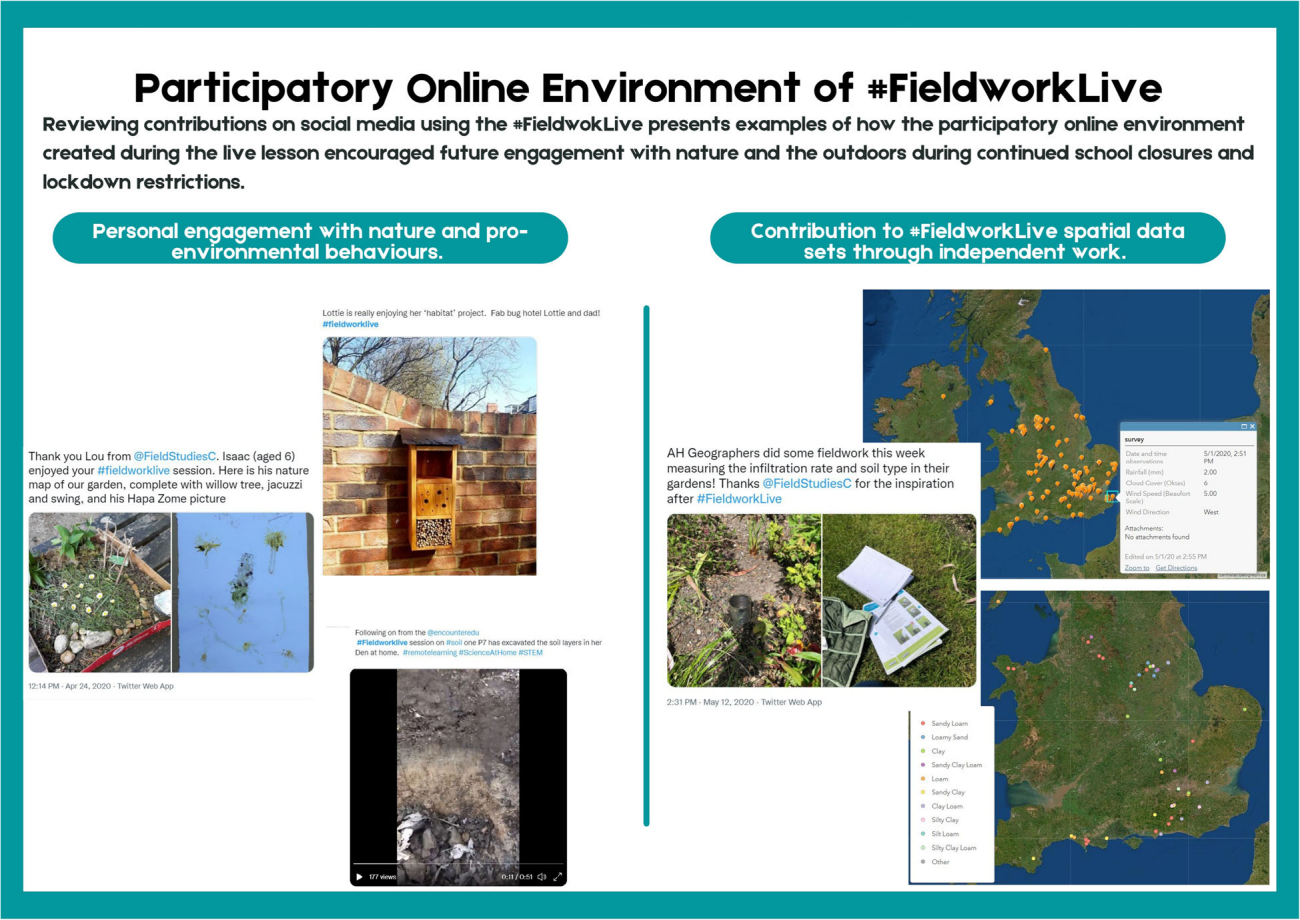
Examples of the participatory online environment created by #FieldworkLive

94% respondents stated that they would recommend the live lessons to other teachers or education providers. 93% described the resource packs as ‘good’, ‘very good’ or ‘excellent’. The reasons why teachers would recommend the live lessons centred around the quality of design and delivery, as well as their role as an educational solution during pandemic lockdowns (Table 1). Teachers especially valued the expertise and personal attributes of the tutors, as well as the tutors’ use of video to demonstrate concepts and exhibit interesting phenomena (Table 2). They also favoured the interactive and practical qualities of the sessions (two examples of the participatory learning opportunities and how they promoted engagement in nature are shown in Fig. 4). 358 questions and 408 ‘shout-outs’ were submitted in advance of live lessons, with a further 16,226 comments and questions submitted using the live chat (Encounter Edu, 2020). The ‘live chat’ function was perceived by some teachers as a positive opportunity for interaction, whilst others found it a distraction (Tables 2 and 3). Similarly, some teachers spoke positively about the content level or pace of the sessions, whilst other criticised these elements. These divergences in opinion might reflect variation in quality and delivery in the sessions attended.

**Table 1:** Emergent themes in the content analysis for response data to the question: “Please let us know the reason for why you would recommend the live lessons to other teachers or education providers” (*n* = 187). Only themes with > 5% respondents are shown

*Emergent theme*	*Percentage of respondents*	*Sample comments*
Enjoyable/engaging/interesting	26	“Live Lessons deepen students’ interest in science study and motivate them” “The information was delivered in a fun way”
Useful/helpful	10	“I found the two ‘A Level Geography’ sessions really useful and worthwhile”
Accessible/user-friendly	13	“Delivered in a very accessible way” “Clear and straightforward”
A valuable form of learning during lockdown	11	“This was a fantastic resource to set for the students, especially during lockdown”
A valuable substitute for fieldwork during lockdown	11	“We normally carry out our own fieldwork in the summer term, so your resources have been great”
Novel resources and ideas that could be incorporated into lessons to enhance the usual approaches	8	“I can use this model with my students to introduce the fieldwork in an actual lesson” “I’ll probably host my own lesson but use your clips to demonstrate the soil sampling” “Useful for including in topics learnt in the classroom”
Fresh perspectives and insights for teachers/CPD opportunity	9	“It helped me as an NQT to develop my fieldwork tool kit” “Gave a good insight” “Great CPD for staff”

**Table 2:** Emergent themes in the content analysis for response data to the question: “What did you like about the #FieldworkLive session” (*n* = 170). Only themes with > 5% respondents are shown

*Emergent theme*	*Percentage of respondents*	*Sample quotations*
General qualities of the presenter	14	“The tutor was very enthusiastic” “Great teachers who the children can relate to, especially [*tutor’s name*] and her tortoises”
Learning from an expert/scientist	6	“Opportunity to see how things are done by an expert” “Was great for students to see experts talking about fieldwork”
Knowledgeable presenter	6	“Knew exactly what she was talking about” “Presenter was clearly knowledgeable”
Appropriate pace/delivery/level of content	12	“Not too long, concise and well explained” “KS2 lessons were very age appropriate”
The visual/demonstrative qualities of the video instruction	10	“Recreation of the coast using the pond and the tiles was creative and helped pupils visualise” “Interesting to actually see the investigation e.g. the types of rocky shore organisms we were shown”
Practical/ ‘hands on’	8	“Good, practical sessions”
Interactive	7	“Interactive and engaging because we could get involved using the SACFOR abundance scale”
The live chat/ability to ask questions	7	“I liked how you could interact with the online teacher and ask questions if you didn’t understand them” “Responses to live comments and questions”

**Table 3:** Emergent themes in the content analysis for response data to the question: “How could the #FieldworkLive sessions be improved?” (*n* = 145). Only themes with > 5% respondents are shown

*Emergent theme*	*Percentage of respondents*	*Sample quotations*
Content issues/additional content ideas	14	“I think they could be a bit longer as they are so good” “His demo of mini-ranging poles and levelling just didn’t work” “More A level focus and content”
Technical difficulties with the live streaming and difficulty connecting	12	“Technical issues - the event did not happen. We had over 100 students geared up and raring to go.” “It didn’t work and was super glitchy and slow.”
Choice of location due to pandemic constraints	12	“It would have been better to be in the location or have videos from the location studied but obviously was not possible with current restrictions.”
Abuse of the ‘live chat’ by students & issues with shout-outs/Q&A	11	“Some inappropriate comments being made.” “Distracting, silly questions.”
Pace/delivery/level of content issues	11	“Pause when the children have to write an answer down and repeat slowly” “More time for pupils to complete the tasks”
Poor sound quality	10	“Hard to hear things at times (busy road in Ipswich).” “The wind made some of [*tutor’s name*]‘s speech very hard to hear.”
Quality of images/video. Additional or alternative images and videos needed.	9	“Some tasks were hard to do with only one image” “Video clips rather than still images would have allowed them to practise the technique more effectively” “More images of places through time - for example the other coastal defences at Slapton or Dedham, not in lockdown”
Logistics/guidance/support for teachers	9	“Tell you how to get the sheets and pre lesson and make sure they know about it before the lesson” “Be clear when resources that were mentioned will be available and specify where to find them”

A significant minority (12%) mentioned technical difficulties that prevented them from joining the live lessons or experiencing them fully (Table 3). A similar proportion specified issues with the audio quality. Other suggestions for improvement included the quality of visual aids used in the videos and the logistical support for teachers.

Many teachers felt that the resource packs provided valuable preparation for the live lessons, or enhanced the live lessons by keeping learners focussed, deepening, and extending learning (Table 4). The ArcGIS StoryMaps were considered to be a particular strength of the resource packs (for example see https://bit.ly/fieldworkliveHydrology). Some teachers thought that resource packs could be improved with additional resources for teachers or students, or by reducing printing requirements (Table 5). One in ten teachers thought that the resources required more differentiation for lower ability students.

**Table 4:** Emergent themes in the content analysis for response data to the question: “What did you like about the resource packs?” (*n* = 188). Only themes with > 5% respondents are shown

*Emergent theme*	*Percentage of respondents*	*Sample quotations*
Enhanced the live lesson by promoting active learning/deepening or extending learning	24	“Referred to directly during the webinar and used in detail, allowing students to be fully focussed throughout” “Helped students get a deeper understanding” “Gave more info for kids who wanted them” “Got us thinking about soil and discussing different types of rock”
Provided valuable preparatory learning for the live lesson	14	“Pre learning is a great way to make the live lessons work and to put context into the session” “Got students thinking about the suitability of the location and hypotheses” “The pre-teach is essential. Don’t think it would be anything like as engaging without it – it preps and motivates students”
The ArcGIS StoryMaps were particularly valuable for learning	6	“Loved the Storymap of Dedham - exposed students to secondary data and how you can create a sense of ‘place’ with a mixture of information”

**Table 5:** Emergent themes in the content analysis for response data to the question: “How could the resource packs be improved?” (*n* = 138). Only themes with > 5% respondents are shown

*Emergent theme*	*Percentage of respondents*	*Sample quotations*
Additional resources for the teacher or students, for example sample answers, glossary	21	“Maybe a glossary of key terminology” “Refer to how each element could be asked in an exam” “Additional links to revision sites for students to complete knowledge retrieval or interleaving from areas they have studied before”
Worksheets in editable format/reduce printing requirements	18	“Provide an editable version so they can be filled in on a computer” “Less ink on the headers of the print outs”
More differentiation for lower ability students	11	“Differentiation. Some of weaker students found it challenging” “Little more straightforward for low ability students”

Survey data identified that an ongoing challenge for organisers was the overly short time frame for design and delivery of #FieldworkLive, coupled with frequent shifts in national policy and constraints on staff availability because of the pandemic, for example:

*Rapid to no handover between those working on resources who were subsequently furloughed and those continuing development.*

These conditions resulted in unavoidable compromises in the design and delivery of the technology enhanced learning:

*We cannibalised already-existing content, rather than be innovative and create new learning tasks from scratch. There was also no time for trialling the Fieldwork Live mode of delivery, and this was not ideal.*

But respondents applauded the team’s ability to adapt to the unusual circumstances:

*The biggest success was how naturally the FSC’s tutors took to online delivery, with the size of the audience surprising all.*

*Ability, resilience and commitment of colleagues to adapt resource development and fieldwork teaching to a digital format.*

### What were the affordances and constraints of this type of technology-embedded teaching and learning, for the delivery of environmental science education during COVID-19? What were teachers’ attitudes to the technology-embedded teaching and learning, and how have these changed because of #FieldworkLive?

‘Closed’ question data from the teachers’ survey indicated that 44% respondents found out about #FieldworkLive directly from the FSC (for example, email and social media) and 56% heard about it from other sources (for example, Encounter Edu, word of mouth, other social media sources). This finding suggests that the events attracted a substantial number of teachers that had not previously accessed the FSC’s educational provision.

In the organisers’ survey, respondents agreed that the most significant opportunity provided by #FieldworkLive was in enabling the FSC to extend its reach and impact:

*Digital delivery offers greater reach and scale allowing for more young people to access high quality tuition from the Field Studies Council.*

*Getting the FSC out to new people in new places, we reached hundreds of students and teachers.*

One respondent added that the removal of cost constraints was a key enabling factor and another described the scaling-up as a highly cost-effective approach. Mass-audience events could be used to promote FSC courses and paid-for digital products, as a way of justifying their cost.

Participants in the organisers’ interview were very clear that large-scale broadcasts were not effective for engaging ‘hard-to reach’ audiences, a persistent challenge for environmental learning providers, without targeted engagement work occurring in tandem:

*We all know that hard to reach audiences are hard to reach for a variety of reasons, not just sort of ease of accessing some technology or opportunities.*

One member described how teachers with low digital confidence may perceive class participation in a broadcast as a daunting or risky undertaking:

*What if I can’t get the technology to work? What if I can’t do 123? What if my school network’s not up to it?*

The four participants in the organisers’ interview expanded on the role of technology enhanced learning in supporting outdoor learning. It helps to prepare and acclimatise learners that have little experience of outdoor environments:

*Those communities who are unfamiliar … their learning in the outdoors is challenged because those experiences are so novel and so new.*

It allows for the development of a teaching model where the elements of learning that do not require an outdoor environment can be completed beforehand. Learners can practise data collection techniques online or in their home locality, rendering them better prepared for the ‘high stakes’ assignment-dependent data collection that they undertake on a residential field trip:

*There is more opportunity to revisit … multiple opportunities.*

*Really thinking about: “Okay, how can we progress individual students, and it’s less high risk, it’s less that one time opportunity to do it”.*

The four respondents in the organisers’ survey thought that the FSC’s customary learner-centred approach had suffered in #FieldworkLive, with less opportunities for learner engagement, interaction, and differentiation than their usual mode of delivery:

*This increased scale can mean a lower level of engagement and interaction for participants with the inability to personalize and differentiate learning.*

*Not bring able to clarify if the students weren’t getting the content. Very scripted and not able to [be] immersive or responsive because students weren’t there with you.*

Respondents also mentioned the lack of formative assessment:

*No verbal feedback or questions.*

*Although some interactivity was built into each session (*i.e. *tasks for learners to carry out at home), there was no way for us to assess that these had been carried out, and how well each learner performed in each task.*

In the organisers’ interview, three participants considered whether these issues were genuinely a consequence of the delivery mode, or the unusual circumstances of the pandemic instead. Most teachers and students were working from home, with a minority (for example, the children of ‘key workers’) in the classroom. Live lessons are normally viewed in the classroom, where the teacher acts as an intermediary, for example inviting the class to pose questions to the presenting scientist or drawing pupils’ attention to notable details:

*You’ve got a classroom of students with the teacher, kind of channelling them into … the live.*

The teacher’s usual role is to evaluate the teacher guidance and pre-lesson resources, identify which elements catered for students’ needs and tailor them accordingly. We were mindful in the design and wording of resources that in some circumstances parents would be required to take on the role of teacher. Preparatory resources were designed to support learning but not to be a pre-requisite, as we knew some learners would not engage with these resources for a variety of reasons. At this stage of the pandemic, schools were at very different stages in terms of their remote learning provision, so it was important to design resources to be used flexibly, and with different levels of teacher input.

In normal circumstances, many of Encounter Edu’s programmes offer differentiation options, for example introductory, ‘refresher’ and advanced broadcasts. Quizzes are used to provide formative assessment:

*You put in a quiz below the video … for learners to get a sense of, you know, are they extracting the right kind of material.*

The three participants discussed how voting tools, polls, ‘live chat’ and discussion boards could improve engagement in live lessons:

*There’s some kind of conduit for the interaction.*

*Being able to collect questions on the live chat and respond to them in the live delivery (and shout outs) increased engagement and understanding. Students - allowed them to engage in a different way and maybe be excited about their own fieldwork in the future.*

One participant emphasised the need to train facilitators in the use of these tools, how to interact remotely with learners during synchronous events, and the signposting and scaffolding that is required to prepare learners for using asynchronous resources.

Another participant believed that such tools could even enhance student and teacher interaction, by providing an anonymous communication medium that is free of the hierarchical constraints found in the classroom:

*Including the kid at the back of the class more because everybody’s on an equal footing.*

Teacher survey data indicated that live lessons had an additional value for teachers, which extended beyond their role as a one-time teaching intervention. A sub-set of teachers described how they had extracted novel approaches or resources from the live lessons, to incorporate into their own lessons (Table 1). Others mentioned the fresh perspective they had gained from the live lessons or described them as an opportunity for professional development.

In the organisers’ survey, one respondent described a teacher-centred pedagogy emerging from participation:

*Some teachers used #FieldworkLive as a platform and jumping off point and then took resources and were able to sign-post/tailor/present the content to their own students. This is something that supported personalised learning.*

Interestingly, in the organisers’ interview two organisers pointed out that the value of #FieldworkLive for teacher development had occurred purely by chance. This part of the feedback may have originated from the teachers that were participating in a FSC event for the first time, who were not familiar with the organisation’s learning approaches, which differ substantially from those typically used in this field of education.

Three respondents in the organisers’ survey believed that teachers’ attitudes and self-efficacy for technology enhanced learning has improved through their participation:

*#FieldworkLive has shown that digital broadcast education can play a meaningful role in teaching and learning, with many more teachers now comfortable with the use of such technologies.*

### (3) What were the advantages of the technologies used by FSC because of COVID-19 that we should continue to practice in and out of classroom learning?

In the organisers’ interview, all participants agreed that large-scale in-field broadcasts would continue to be a valuable approach beyond the pandemic, for introducing new audiences to outdoor science education:

*Joining something that’s easy to join in, as a first step.*

*There’ll be teachers who are … really confident with digital type things, but who wouldn’t want to take their children out of a classroom.*

Nature broadcasts were perceived as a valuable brief intervention for stimulating teachers’ and students’ interest in outdoor science, or one that could be used to provide study support at critical points, for example leading up to exams. Participants discussed a variety of benefits of this approach, many of which focussed on access and inclusivity. Broadcasts allowed learners to experience ecosystems or experts that they would not normally have access to, either due to geographic location or cost:

*We also have to look at the fact, and be quite critical, that outdoor learning has served a certain group of school learners.*

*I’m going to give you access to an amazing rocky shore environment, because you live, you know, how many 100 miles away from that.*

*We want to give you access to this amazing speaker, to this amazing place.*

One participant emphasised that technology enhanced learning should not be seen as an inferior approach to outdoor learning for reaching these under-served schools, and should be designed to provide an equivalent experience, with similar opportunities for progression.

*We have to be careful that we don’t have a “outdoors is better, digital is the poorer sister”.*

Three participants agreed that the ‘wrap-around’ blended learning approach, where digital resources provide the preparation and follow-up learning for outdoor science, poses certain risks. The FSC recently adopted this blended learning approach and the model requires refining. A teaching model that is reliant on schools completing preparatory learning could fail to deliver on the targets set for the outdoor science delivery, if preparatory tasks have not been completed. Field centres may not know whether to prepare introductory field activities, or activities that follow on from the preparatory learning, for a particular school:

*You might try and push some of the teaching and learning … into an alternative mode of delivery that might be digital … But that bit in the middle is very reliant on the bit at the start.*

*If you send out pre course stuff, you don’t know how well they’ve taken it, or how much they’ve covered And have they done it in the way you thought they were?*

Three members agreed that a preferable blended learning model was one where the digital learning was part of an iterative process, more akin to an individual learner’s journey, instead of as separate components that occurred in a fixed order. One participant suggested that this model could offer different approaches or contexts for developing a particular skill, thus allows learners to reach specific outcomes whilst tailoring learning and/or practising the skill under different conditions. Boundaries between the digital and outdoor components were less defined and offered choices for the route and order that learners took through the content. Another participant compared this approach to a ‘choose your own adventure’ book, because it allowed learners to branch off in different directions.

*So, I think when we’re thinking about blended, it has to be truly blended, and not just: “here is your intro prep that you do at home before you come”; field work, and then some follow up. But actually, it’s a way of revisiting various different opportunities. So that it’s more akin to an individual learner’s journey, perhaps.*

*It could be quite easy for us as, as people who are used to working in the outdoors … to prescribe a route through.*

*A selection box from which students and teachers and schools can select the bits that work best in their context.*

*I think it’s about having flexible routes through things … ways of revisiting, going back over different scenarios, but the same skills, for example, and applications, in different contexts. So that it is still progression, but there are multiple ways of achieving those skills or development.*

*If you want to find out more about this because you need to work on this element, click here and it takes you to some other section... tree sort of approaches where you branch off and dip into that if that’s what you need but actually not everyone needs to go there.*

All participants agreed that the technical constraints associated with #FieldworkLive could be overcome, with a modest organisational investment. One participant also highlighted the importance of factoring learners’ technical constraints into future digital design, for example the huge variation in size and specification of computers that learners were using. Priority areas for financial investment were audio equipment and data-only mobile hotspots with an ample data allowance, to use in locations with poor connectivity. Two participants highlighted the need to assess future filming locations for internet connectivity and ambient sound issues, in addition to their educational potential:

*You now have time to … go into various interesting places in your field centres and look at the mobile strength.*

*It would be great to have, you know, multiple camera views and a higher quality camera: but actually, would that add that much for the cost? But something like, you know, better sound equipment? Yes, that wouldn’t be as much but would add a lot.*

*Sound’s always more important than video: always, always, always.*

Participants discussed the value of static images and pre-recorded segments in broadcasts, both as a way of overcoming unpredictable aspects of the environment and for enriching the educational quality with minimal cost:

*If it’s inside a dairy shed, and the cows might be behaving or not behaving then that is shot and then zoomed out from some studio setting with an ethernet cable.*

*To be able to show some of those stills, or to talk over the top of a worksheet, those sorts of things made a huge difference.*

## Discussion

#FieldworkLive provided a valuable learning opportunity for FSC staff and Trustees, alongside the other digital programmes produced for schools during COVID-19, which included an online science festival, virtual fieldwork packages, teacher CPD webinars and a series of live broadcasts for primary schools. FSC’s location-based residential and day trips for schools have now resumed but there has been a permanent shift to a blended learning model alongside the in-person delivery. This development could allow FSC to broaden its reach and inclusive provision as part of its charitable objectives, as well as providing a more progressive fieldwork experience. Another key impact that the pandemic had on ethical practice and equity, was the production of an organisational risk assessment and safeguarding policy for digital learning (whereas Encounter Edu’s policies were used for #FieldworkLive).

#FieldworkLive appears to have been a successful programme of learning, as demonstrated by the high rate of participation and subsequent feedback. Teachers described the live broadcasts and associated learning as interesting, engaging, and worthwhile. A key reason for #FieldworkLive’s efficacy was the calibre of the presenting tutors, with many teachers praising their expertise and personal attributes. This echoes the findings in other studies, that the quality of the pedagogy was more important than the technology employed (Fletcher et al., 2007) and that video instruction was more effective when instructors were enthusiastic and affable (Guo et al., 2014).

#FieldworkLive provided secondary schools with a much-needed fieldwork substitute and online learning provision during the lockdown restrictions. But the value of this digital learning approach was not just in providing a timely intervention during a challenging time. Some teachers (particularly recently qualified ones) recounted that participation had developed or enhanced their professional skills. Teachers as well as students gained access to a team of experts, through the broadcasts, the question-and-answer sessions, and guided resources. Kulgemeyer (2020) highlighted the value of instructional, expert-led science videos for both teachers and students. The live broadcasts enabled teachers to develop their pedagogical content knowledge, by observing novel approaches for teaching techniques and concepts (Fig. 5)*.*

**Fig. 5:**
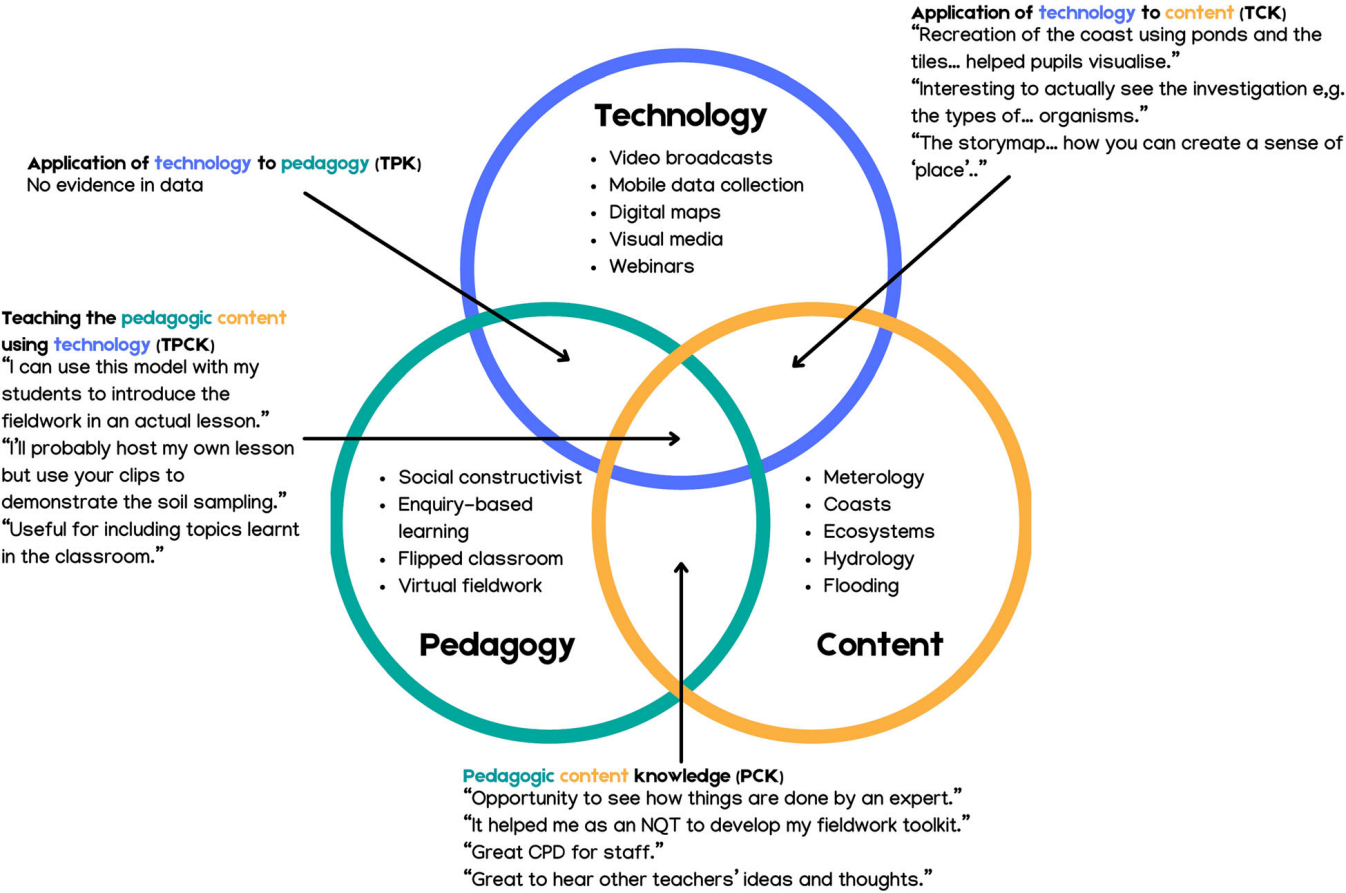
Emerging model for the affordances provided by this technology enhanced approach, based on Koehler and Mishra’s (2005) framework (quotations are derived from teacher feedback data)

Other teachers explained how their participation had developed skills in technology enhanced teaching and learning, a trend that was also noticed by one of the organisers (questionnaire data). They specified elements of #FieldworkLive that they planned to extract for use in future lessons, or novel approaches that they intended to apply. In this way the live broadcasts and online archive of content contributed to teachers’ technological pedagogical content knowledge, about using technologies in constructive ways to teach content (Mishra & Koehler, 2006) (Fig. 5)*.* Teachers’ low confidence in embedding technology in teaching is well documented (for example, Bingimlas, 2009) and is a key factor for the disconnect between students’ experience of technology inside and outside of school (Wang et al., 2014). Our findings contribute to the development of the TPCK framework by providing a practical example of its application in science and geography education. Voogt et al.’s (2013) review of the TPCK literature highlighted the scarcity of experimental evidence specific to subject domains.

These emerging affordances demonstrate the potential value of a flexible package of multimedia resources that teachers and learners could adapt to their own needs, as proposed in organisers’ feedback. It is an approach that takes account of the complexity of the educational settings in which technology integration occurs and focuses on learning processes, instead of just providing a novel instructional activity (Hamilton et al., 2016). It is also compatible with mobile learning, which occurs across a range of informal settings in time and space (Kearney et al., 2012). Jacobson et al. (2009) found that a flexible, extensible, non-linear fieldwork package about soils and civilisations was effective for promoting active learning and critical thinking. As a result, FSC is now developing a compendium of short video, text, graphics, and map-based resources for schools from the substantial digital content produced during the pandemic (https://www.field-studies-council.org/digital-hub/). Several studies have shown that short instructional videos were more effective than long ones (for example, Guo et al., 2014).

Another importance affordance of large-scale live broadcasts is their efficacy in engaging new audiences, as evidenced in the teacher and organiser data. The organisers agreed that such field-based broadcasts should continue to be used, as an engaging and accessible introduction to outdoor science education, whilst acknowledging that this approach could not address all the barriers currently preventing participation. We do not know what proportion of participating schools were in areas of high deprivation or marginalised communities, so would aim to investigate this in future programmes. Virtual field experiences enriched science learning for students excluded from field trips by familial or financial circumstances (Shinneman et al., 2020) or by disabilities (Clark & Jones, 2011; Atchison, Marshall & Collins, 2019). They can provide experiences of environments that are inaccessible, due to remoteness, fragile or hazardous features (Stokes et al., 2012). They also allow students to experience environments at temporal or spatial scales that cannot be observed directly (Jacobson et al., 2009).

Video instruction was shown to have a particular role in #FieldworkLive for demonstrating field skills and displaying biological or environmental phenomena to learners. Welsh et al.’s (2013) survey of fieldwork practitioners identified that a key pedagogic reason for integrating technology into fieldwork was to develop students’ subject-specific and transferrable skills. Video instruction in science helped to convey the cultural, visual, and auditory qualities of the location (Jacobson et al., 2009) and supported conceptual learning through the development of memory cues and connections (Eick & King Jr, 2012). Video is an educational medium that is effective with teachers and students alike, as both target audiences regularly use video-sharing sites out-of-school (Wang et al., 2014; Bardakcı, 2019).

Science videos are enhanced through the addition of static images for detailed features, which in #FieldworkLive was mainly used to support learning about data collection and analysis. Organisers and teachers highlighted issues with video production quality that were mainly due to pandemic constraints and could be overcome in the future with a modest organisational investment. Production quality is a common limiting factor on the pedagogic value of video in educational settings (Laaser & Toloza, 2017). Animation was a valuable addition to videos for illustrating abstract scientific concepts and phenomena that cannot be viewed directly in Jacobson et al. (2009) and in the first author’s previous work (https://www.futurelearn.com/courses/future-food).

In questionnaire feedback, organisers agreed that the most unsuccessful aspect of this scaled-up digital approach was the limited extent of learner engagement, interaction, and differentiation. Barton’s (2020) study of pandemic field instruction in the university sector found a similar result, with instructors’ expressing negative views about digital substitutions as being less active and less learner-centred than field ones. In the group interview, organisers considered these unsuccessful aspects to be mainly due to the pandemic restrictions, rather than constraints posed by this type of technology enhanced learning. Authors have highlighted interactivity as a particular strength of live video-streaming, compared to other digital formats. Students and teachers can communicate directly with presenting scientists (Scowcroft et al., 2015) and even influence the direction and focus of a scientific enquiry (Robson et al., 2017). Organisers identified a variety of digital tools and strategies for improving learner interactivity in the synchronous and asynchronous modes and highlighted the need for coaching instructors in using these approaches. Teachers highlighted that resource packs could have been better designed to highlight differentiation opportunities for lower ability learners.

In teacher feedback, some respondents praised the interactivity of #FieldworkLive, although opinion was divided about the value of the tools and approaches that were employed, and some felt that the design and moderation of these needed to be improved. Students were working from home, away from the regulated, classroom environment; this age group has a known propensity to use digital media for social interactions (Bolaños & Salinas, 2021; Thompson, 2013). Gewin (2020) highlighted the importance of including regular opportunities for learner interaction and feedback in technology-embedded learning, as part of good practice. The number of questions submitted during live lessons was considerable, which presented a challenge for the responding tutors. An alternative with large audiences is to use voting polls instead, which Cooke et al. (2020) used as a way of engaging remote students in designing actual fieldwork, and to support schools in follow-up work for reflection and consolidation.

Live lessons were designed to promote active participation by linking to practical exercises and investigative work, which many teachers highlighted as a positive feature. The resource packs were an essential element for the active learning approach, and teachers commented on how the resources helped to provide context to the live lesson, increased learner motivation and interest, and encouraged reflection and deeper learning. Other authors have commented on the importance to scaffold learning from video using supplementary resources (Burden & Atkinson, 2007; Bardakcı, 2019).

Organisers felt that teachers’ attitudes towards technology enhanced teaching had improved because of their participation in #FieldworkLive (questionnaire data). There was no direct evidence of attitudinal change from teacher data, but we can assume that the impact on attitudes was positive for the many teachers that described the experience as accessible and user-friendly. #FieldworkLive’s reliance on familiar technology provided high usability, a crucial element for technology-embedded learning, since both teachers and young people are known to have limited digital skills (Bingimlas, 2009; Bolaños & Salinas, 2021; Wang et al., 2014). Gewin (2020) emphasised the importance of not relying on live conferencing as a sole delivery medium due to the risk of unforeseen technical problems.

Some teachers felt that the logistical guidance and support for teachers could have been improved or were unhappy with the pace of delivery or level of content. A significant minority of teacher experienced technical problems that prevented them from participating (or participating fully) in #FieldworkLive. These experiences could have impacted negatively on attitudes to technology enhanced learning, which is considered risky or unreliable by some educational practitioners (Welsh et al., 2013). Organiser interview data indicated that most technical faults could be overcome in the future, with additional investment and planning.

This study has provided a reasonable picture of the attributes of the technology enhanced learning employed (research questions 1 and 2) but only a partial understanding of teacher’s attitudes and the advantages of the technologies that we should continue to use (research question 3). A key limitation of this study was the brevity of the teacher evaluation, which could have been designed to capture a more extensive picture of teachers’ attitudes and perceptions (through the inclusion of video interviews, for example). The constraint on time for adequately piloting the teacher survey meant the questions could not be refined based on the dominant themes in a preliminary dataset, which explains the low frequency for many of the emergent themes. The lack of an internal review immediately after the programme delivery, and lack of student evaluation, were also missed opportunities to gain deeper insights into the mechanisms of blended learning. Like the programmes themselves, monitoring and evaluation instruments had to be designed and delivered in haste, due to the unusual circumstances of the pandemic. Another limitation of the data collection was that the group interview with organisers tended to drift to a discussion of the general advantages of digital technologies for outdoor learning, rather than maintaining a focus on the technologies used during COVID-19. This outcome demonstrates the importance of concise questioning and strong facilitation during such sessions.

## Conclusions

This study provided novel insights about the role of live broadcasts and video-based instruction in curricular science, geography and outdoor learning. A key affordance of the live broadcasts was their ability to engage new audiences, by providing an engaging and accessible introduction to relevant topics and techniques. Participation in #FieldworkLive was shown to contribute to teachers’ technological pedagogical content knowledge and pedagogical content knowledge. These findings contribute to the development of the TPCK framework by providing a practical example of its application in science and geography education. Video-based instruction was a valuable medium for learning topics and techniques, when accompanied by written instruction and student tasks. Video instruction presented challenges for learner engagement, highlighting the importance of training facilitators in the use of digital tools and approaches that increase interactivity with learners.

FSC, like many organisations, underwent a digital transformation during the pandemic, with a rapid development in staff skills and expertise, as well as a programme that reached a large, diverse audience. As a result, FSC is broadening to a blended learning delivery mode, with associated pedagogic and inclusivity benefits.

## Supplementary Information

**Additional file 1: Table S1.** Questions for online teacher survey. **Table S2.** Questions for online organiser survey. **Table S3.** Questions for online organiser discussion group. **Table S4.** Analysis of organiser questionnaire data. **Table S5.** Analysis of organiser questionnaire data (SE = Provided significant statement or example suggesting agreement, NR = Did not indicate agreement or dissent (i.e., nonresponse).

## Data Availability

The datasets used and analysed during the current study are available from the corresponding author on reasonable request.
