# Supplementary material for: Expanding the field: using digital to diversify learning in outdoor science
Source: Discip Interdscip Sci Educ Res. 2022 Mar 10;4(1):9. doi: 10.1186/s43031-022-00047-0 (PMC8908296; doi:10.1186/s43031-022-00047-0)
Supplement: Supplementary file 1 — Additional file 1: Table S1. Questions for online teacher survey. Table S2. Questions for online organiser survey. Table S3. Questions for online organiser discussion group. Table S4. Analysis of organiser questionnaire data. Table S5. Analysis of organiser questionnaire data (SE = Provided significant statement or example suggesting agreement, NR = Did not indicate agreement or dissent (i.e., nonresponse). [file 43031_2022_47_MOESM1_ESM.pdf]

Appendices

An online questionnaire was circulated to participating schools immediately after #FieldworkLive took place using and was completed by 233 teachers from 26 April to 11 May 2020. The questionnaire featured the questions in Table 1.

Closed questions	*Open questions*
1. How did you find out about the FSC #fieldworkLive events?<br>2. Would you recommend the live lessons to other teachers or education providers?<br>3. How useful did you find the resource packs for pre-lesson work and teacher guidance?<br>4. Which #fieldwork live session did you attend?<br>5. How much progress do you think your students will make after taking part in a #fieldworklive session and completing the corresponding student activities?<br>6. Would you use these if available?	1. Please let us know the reason for your answer to 'Would you recommend the live lessons to other teachers or education providers?'<br>2. What did you like about the resource packs?<br>3. How could the resource packs be improved?<br>4. What did you like about the #fieldworklive sessions?<br>5. How could the #fieldworklive sessions be improved?<br>6. If we were to charge for fieldwork live in the future, how much do you think your school would be willing to pay for access?<br>7. What would you like to see in our digital product offering?<br>8. Do you have any other comments you would like to share with us?

Table 1. Questions for online teacher survey

Six #FieldworkLive organisers (including the third author) participated in an internal consultation from 15 July to 16 September 2021. The consultation included an online questionnaire with six open-ended questions about organisers' perceptions of the technology-enhanced learning (Table 2) and a one-hour group interview by video conference about the educational implications of the technologies used, which was guided by five leading questions, based on the key emerging themes in the questionnaire data (Table 3). The data analysis for the online questionnaire is shown in Table 4 and the data analysis for the group interview is shown in Table 5.

What were the educational opportunities and constraints associated with the format and delivery mode of #FieldworkLive, compared to our usual 'in-person' delivery?
What were the biggest challenges, in designing and implementing #FieldworkLive?
What were the most successful aspects of #FieldworkLive? What do you think we should do differently in the future?
What were teachers' and students' attitudes and reactions towards the technology-enhanced teaching and learning?
What impacts do you think participation in #FieldworkLive has had on students and teachers?
Were there any advantages of the digital technologies or approaches we used during Covid-19 that we should continue to apply in FSC in the future?

Table 2. Questions for online organiser survey

Everyone agreed that #FieldworkLive allowed FSC to reach a larger and broader audience. **Does this opportunity still apply, with the return to fieldwork, and if so, how should we use or design TEL to maximise our reach, particularly to under-represented audiences?**
Most people thought that the most important role for technology enhanced learning in FSC was as a 'wrapper' for fieldwork. **Are there any disadvantages of this approach and what considerations are there for design?**
Everyone agreed that the biggest weakness of this delivery mode was the limited opportunities for student interaction, differentiation, and assessment, compared to in-person modes of delivery. **Is it possible to address these constraints through design, or is it an unavoidable constraint?**
#FieldworkLive posed some technological challenges, for example the high take-up, which crashed EE's servers, the high bandwidth connection needed for delivery of online lessons, buffering on the pre-recorded sections, poor audio recording. **How could these challenges be addressed in the future, within budgetary limitations?**
Teacher feedback indicated that a key factor for #FieldworkLive's success was the expertise and enthusiasm of the presenting tutors, the quality of the accompanying resources, and the fresh perspectives it provided, about fieldwork. **How should these opportunities be developed and explored in the future?**

Table 3. Questions for online organiser discussion group

Expanding the field: using digital to diversify learning in outdoor science - Learning/Unlearning during the Covid-19 Pandemic: Futuristic Science Education

**Theme**	**Relevant statements in questionnaires (colour denotes respondent identity)**<br>**Respondent 1 – red. Respondent 2 – blue, Respondent 3 – purple, Respondent 4 – green, Respondent 5 – brown.**
#fieldworklive allowed the Field Studies Council to extend its reach and impact	Getting the FSC out to new people in new places, we reached hundreds of students and teachers.<br>Connecting with new and wider audiences.<br>Present FSC fieldwork delivery to a wider/broad audience as cost constraints were removed.<br>Digital delivery offers greater reach and scale allowing for more young people to access high quality tuition from the Field Studies Council.<br>This additional scale is also cost-effective
Tutors were not able to apply their usual learner-centred approach due to the constraints on learner engagement, interaction, formative assessment and differentiation	Not being able to clarify if the students weren't getting the content. Very scripted and not able to immersive or responsive because students weren't there with you.<br>No verbal feedback or questions.<br>There was no way of focussing the participants on the work as they were lone working (not with their teachers) and too distracted by writing stuff on the live chat.<br>Interaction was limited. Not able to perform any formative assessment and respond to needs of learners. Pace was fixed.<br>However, this increased scale can mean a lower level of engagement and interaction for participants with the inability to personalize and differentiate learning.<br>I think that the lack of interaction between teacher and learner was the biggest shortcoming of the mode of delivery.<br>Assessment and differentiation were very limited as a result.<br>A skilled teacher (including our centre tutors) will be continually assessing leaners' progress throughout, from assessing prior learning at the start of the session, formative assessment at regular intervals (e.g. with a plenary after each task) and some form of assessment at the end.<br>Although pre-session tasks were given, we didn't check that these had all been completed, so learners could have arrived at the session with a wide range of prior learning.<br>…although some interactivity was built in to each session (i.e. tasks for learners to carry out at home), there was no way for us to assess that these had been carried out, and how well each learner performed in each task.<br>At worst this could have led to an experience for learners which was as passive as watching a televised sports match while lying on the sofa.
The pandemic presented a number of challenges to #fieldworklive design and delivery	Maintaining agile and responding to changes in national policy.. Exams Cancelled for 2020… Stay at Home Order… Furlough introduced.. *[goes onto describe FSC's adaptations to each challenge]* First time that most colleagues had worked remotely, online. Rapid priority change from 'day job' to 'development'. Working without existing internal line manager/support structures, in a new team._<br>Practical constraints associated with a small development team (2 writers and a designer) and limited development time (10 days).
The pandemic-related challenges led to some unavoidable compromises in design and delivery of #fieldworklive	Leap of faith needed in the concept/delivery mechanism/theme without the content being developed, as there was a need to advertise it to schools prior to having a full-understanding of what it entailed.

	Not all colleagues were able to fully-get on board with rapid digital development in the short timescales involved..<br>But these constraints meant that we cannibalised already-existing content, rather than be innovative and create new learning tasks from scratch.<br>There was also no time for trialling the Fieldwork Live mode of delivery, and this was not ideal.
The FWL team were competent in adapting to the unusual circumstances	Ability, resilience and commitment of colleagues to adapt resource development and fieldwork teaching to a digital format.<br>The biggest success was how naturally the FSC's tutors took to online delivery, with the size of the audience surprising all.<br>The event sustained the FSC's status as the leading organization for fieldwork and fieldwork skills provision.
Digital learning could play a valuable role in the future as a 'wrap-around' for fieldwork	Pre- and post- work of more value by using tech.<br>Blended learning - preparing them using StoryMaps - this has been bought into the GCSE standardisation as a formula to prepare students for fieldwork.<br>Use of digital fieldwork resources to provide wrap-around support for fieldwork experience, a true blended fieldwork approach.<br>Build progression in skills and confidence prior to in-field fieldwork.<br>Opportunity for multiple low-stakes fieldwork opportunities, rather than the high-pressured high-stakes residential or day fieldwork opportunity.<br>Use of teacher support (Guidance Docs/Webinars) to better support teacher pre and post fieldwork experience with FSC.<br>With the aim to better integrate fieldwork within classroom<br>When assessing the delivery methods to be used by FSC in the future, a blended approach seems appropriate<br><br>Small-group online (e.g. delivered by Zoom or MS Teams): could be used as prep of [*or*?] follow-up work from in-person visits..
Teachers' attitudes and confidence in digital learning has improved as a result of their participation	Teachers - made them more aware of the possibilities (and maybe the FSC?).<br>Greater awareness of how fieldwork can be enhanced/supported through digital methods.<br>However, #FieldworkLive has shown that digital broadcast education can play a meaningful role in teaching and learning, with many more teachers now comfortable with the use of such technologies, and providers such as the FSC no longer needing to explain what is involved and how this can be accessed.

Table 4. Analysis of organiser questionnaire data

Emerging theme/assertion	*Respondent 1*	*Respondent 4*	*Respondent 5*	*Respondent 6*
**Technology-enhanced learning is a valuable approach for engaging new and diverse audiences in outdoor science education**	SE<br><br>(Later in the dialogue – around 37 mins)	SE<br><br>Large-scale broadcasts are not effective for engaging 'hard-to reach' audiences alone;	SE<br><br>TEL should not be regarded as an inferior approach to outdoor learning. It is important that TEL provide equivalent experiences to	SE<br><br>FSC and similar organisations have struggled to broaden their reach to new audiences in the past

		additional, targeted work is required<br><br>Large-scale broadcasts are not perceived as a 'low-risk' digital learning option by teachers with low digital confidence	outdoor ones, with opportunities for progression	
**Specific benefits of large-scale in-field broadcasts, or other TEL approaches in outdoor science**	TEL can be prepared in advance, can draw on experts and can be designed for inclusivity more easily than outdoor learning	Broadcasts introduce learners to ecosystems/environments that would not normally be accessible<br><br>May encourage students and teachers to ask more questions (compared to in-person), as broadcasts provide an anonymous communication medium, outside of the hierarchy of the classroom environment<br><br>A class can access materials multiple times e.g. watch a video again	TEL allows us to reach audiences that have been unable to access our fieldwork provision in the past ('underserved' audiences)<br><br>TEL provides a valuable 'stepping stone' to outdoor science learning, particularly as preparatory activities for learners with little outdoor experience<br><br>TEL allows learners to practise data collection techniques digitally, ahead of their assignment-related field data collection	The addition of TEL to FSC's existing offer enables us to reach a more diverse audience, by providing engagement opportunities for teachers with low confidence in outdoor delivery but high confidence in digital delivery and vice versa<br><br>TEL allows us to develop a teaching model where the elements of learning that do not require an outdoor environment can be completed beforehand
**There are challenges posed by a 'wrap-around' blended learning approach where the TEL is used to provide preparatory or follow-up learning for outdoor science**	SE<br><br>Field tutors do not how much preparatory learning a visiting school has completed<br><br>It is a novel approach that has not been refined	NR	SE<br><br>In a blended learning model, the TEL should not be designed as separate components before and after the fieldwork but as part of an iterative process, more akin to an individual learner's journey	SE<br><br>A teaching model that is reliant on schools completing preparatory learning could fail to deliver on the targets that are set for the outdoor science delivery
**An alternative TEL approach to the 'wrap-around' is a flexible blended learning package that allows teachers and learners to**	SE<br><br>Resembles a 'choose your own adventure' book because it allows	SE<br><br>It is more teacher-led and student-led than a 'fixed' programme of learning	SE<br><br>Flexible routes through the content, e.g. offer different	NR

**personalise learning to their own requirements**	learners to branch off in different directions		approaches or contexts for developing a particular skill.<br>This allows learners to reach specific outcomes whilst tailoring learning and/or practising the skill under different conditions	
**Large-scale in-field broadcasts pose a challenge for student interaction, differentiation, and assessment – but it is possible to overcome these challenges**	NR	SE<br><br>The broadcast would normally be viewed in the classroom, where a teacher is present to support learning<br><br>There would often be multiple broadcasts for a particular topic, defined by level e.g. introductory, advanced<br><br>Quizzes can be used for formative assessment, following videos that are accessed asynchronously<br><br>The flexibility and appeal of long broadcasts could be broadened by designing them so that some elements can be viewed separately. Allows more rapid access to the ‘choice’ stage, in the ‘choose your own adventure’ analogy e.g. definitions of technical terms	SE<br><br>Facilitators need to receive training in (1) interacting remotely with learners during synchronous events, and (2) providing signposting and scaffolding to prepare learners for using asynchronous resources<br><br>[Agrees with previous respondent] Need to employ tools for developing an online learning community for both synchronous and asynchronous approaches e.g. polls, discussion boards<br><br>The digital tools or pedagogies that facilitators adopt is determined by size of group and delivery mode (synchronous/ asynchronous)<br><br>Need to guide teachers in how to support their learners whilst accessing digital resources	SE<br><br>The interactivity can be improved by allowing students to influence the direction or content of the broadcast through polls and other voting tools e.g. OU Fieldcasts<br><br>[Agrees with previous respondent] When viewed in class teacher can be the intermediary that provides the differentiation
**The technical constraints in FieldworkLive could be addressed for the future, with a**	SE	SE	SE	SE

**reasonable investment of time and money**	Does not matter production is not too polished; important for viewers to have an authentic experience of the field environment you're broadcasting from	Research potential filming locations, so that you know how reliable the existing connection is and what the limitations are<br><br>Invest in data-only mobile hotspots with a pay-as-you-go SIM with ample data allowance for locations with poor connectivity<br><br>Insert pre-recorded segments into broadcasts, for scenes that you know will need multiple retakes<br><br>[Agrees with previous respondent] audio matters more than video, unless you are presenting a specifically visual element (and you can get round that issue by sending the producer a photo or video in advance of filming)	Video equipment was fit for purpose but investment in better sound equipment would be beneficial<br><br>TEL design should be based on how learners will be using the digital resources and how they will access them (eg type/size of device)<br><br>Invest more time in the design of the accompanying resources, as these substantially enriched the video-based instruction<br><br>Invest in staff – training in video-enhanced delivery as it is very different to in-person teaching<br><br>Need to acknowledge the 'hidden' skillset/time contribution of tutors' partners to FieldworkLive	Visual aids e.g. photos shown on screen were very effective and not costly<br><br>It may be possible to choose more suitable filming locations in the future, without pandemic-related constraints
**The role of FieldworkLive as 'teacher CPD' happened purely by chance**	NR	NR	SE<br><br>It hints at a disconnect between what FSC does and what schools either need, want or expect	SE<br><br>Would be valuable to know if the CPD feedback came from existing customers or teachers that were new to FSC (if the latter could be because FSC's fieldwork approaches are different to the traditional ones, even if well-used internally)

**The educational system is not nurturing young people's digital skills**	NR	SE<br><br>Digital platforms that provide young people with a communication/creative outlet are very popular. But questions whether the educational system e.g. assessment modes are providing young people with appropriate outlets for digital creativity	SE<br><br>Young people's digital skills development during the last 18 months has not been properly evaluated, meaning we do not know the important areas to address<br><br>Authentic assessment using digital methods is becoming more popular in HE and WBL (but still just case studies/pilots)	SE<br><br>Young people's digital skillset is determined by their usage, which centres on social communication rather than education<br><br>Both young people and adults are 'digital natives' but are 'indigenous' to different 'countries'

Table 5. Analysis of organiser questionnaire data (SE = Provided significant statement or example suggesting agreement, NR = Did not indicate agreement or dissent (i.e., nonresponse)
